# Processing tracking in jMRUI software for magnetic resonance spectra quantitation reproducibility assurance

**DOI:** 10.1186/s12859-017-1459-5

**Published:** 2017-01-23

**Authors:** Michał Jabłoński, Jana Starčuková, Zenon Starčuk

**Affiliations:** 1 Institute of Scientific Instruments of the CAS, Královopolská 147, 612 64 Brno, Czech Republic; 2 Faculty of Science, Masaryk University, Kotlářská 267/2, 611 37 Brno, Czech Republic

**Keywords:** Magnetic Resonance Spectroscopy, Signal Processing, SQL database, jMRUI

## Abstract

**Background:**

Proton magnetic resonance spectroscopy is a non-invasive measurement technique which provides information about concentrations of up to 20 metabolites participating in intracellular biochemical processes. In order to obtain any metabolic information from measured spectra a processing should be done in specialized software, like jMRUI. The processing is interactive and complex and often requires many trials before obtaining a correct result. This paper proposes a jMRUI enhancement for efficient and unambiguous history tracking and file identification.

**Results:**

A database storing all processing steps, parameters and files used in processing was developed for jMRUI. The solution was developed in Java, authors used a SQL database for robust storage of parameters and SHA-256 hash code for unambiguous file identification. The developed system was integrated directly in jMRUI and it will be publically available. A graphical user interface was implemented in order to make the user experience more comfortable. The database operation is invisible from the point of view of the common user, all tracking operations are performed in the background.

**Conclusions:**

The implemented jMRUI database is a tool that can significantly help the user to track the processing history performed on data in jMRUI. The created tool is oriented to be user-friendly, robust and easy to use. The database GUI allows the user to browse the whole processing history of a selected file and learn e.g. what processing lead to the results, where the original data are stored, to obtain the list of all processing actions performed on spectra.

## Background

Proton magnetic resonance spectroscopy (MRS) and magnetic resonance imaging (MRI) are non-invasive measurement techniques utilizing the phenomenon of nuclear magnetic resonance (NMR) for detecting signals of hydrogen protons in the human or animal body. Protons, endogenous and abundant in all tissues, provide structural and functional information by probing their nearest biochemical neighborhoods and reporting their positions by responding to gradient-field encoding. It is primarily MRI that is widely used in medicine for diagnosing cancer, injuries, vascular abnormalities, dementia and other diseases [1]. MRI also serves for monitoring disease progression, therapy, and – often as a quantitative tool – for biomedical research. Images, carrying statistical information on local nuclear magnetic interactions, translational motion a random mobility of water molecules, on tissue perfusion, blood oxygenation, or water/fat content, are well accepted by the medical and biomedical users because of their comparability with other imaging modalities, good signal-to-noise ratio, often self-evident verifiability of the absence of artifacts, and reasonable acquisition times. Only in cases of doubt or lack of specific markers, or for specific research do MR technology users add MR spectroscopy to their exploratory portfolio and then often find themselves overwhelmed by unexpected complexity of physics, engineering and data processing connections. Measurement parameter setting, quality assessment and data processing typically require expert knowledge not readily available in radiologists, and unless an experienced MR spectroscopist is available on site, the possibility of documenting all operations, availability of records for consultation, result verification and standardization becomes important.

Unlike MRI, MR spectroscopy, practiced as single-voxel or multi-voxel 1D spectroscopy or 2D or 3D spectroscopic imaging, provides spatially localized information about the concentrations of up to about 20 low-molecular-weight metabolites [2], participating in numerous intracellular biochemical processes. Such information may, for example, provide the physician more specific clues for staging and grading of a tumor previously identified by MRI; the improved assessment of the degree of malignity and prognosis may then contribute to optimizing the therapy. Besides diagnostics of brain tumors, MR spectroscopy has been used in studies of metabolic changes in brain tumors, strokes, seizure disorders, Alzheimer's disease, depression and other diseases affecting the brain and muscles [3]. Depending on the metabolic marker under observation, a suitable MRS acquisition method (pulse sequence) and corresponding data processing must be chosen. For example for the detection of neurotransmitters GABA and glutamate, MEGA-edited PRESS may be preferred [4], while for obtaining information about as many metabolites as possible, the PRESS [5], STEAM [6], LASER [7], semi-LASER [8] or SPECIAL [9] pulse sequences with short echo times could be used; such a choice impacts on the optimal data processing workflow.

Despite the potential of MR spectroscopy in medicine and research [10], its practical usage is still limited. The main obstacles are the facts that spectroscopy itself does not produce an image that can be relatively easily interpreted, signal artifacts are not always easy to recognize, and that to obtain metabolite concentration estimates, the signals have to be analyzed with a sophisticated model fitting (quantitation) algorithm, whose reliability depends on many factors. As the properties of the acquired signals depend on the acquisition method used, the fitting algorithm always needs some specific prior knowledge (such as the signal or spectrum pattern for each metabolite) and constraints. Accurate quantitation of metabolite concentrations is often further complicated by regular or random signal artifacts [10], for which no useful model may be available and which may reduce the credibility of all results if they are ignored. The artifacts become particularly difficult to manage in combination with the fast-decaying signals of macromolecules and lipids, found in signals acquired at short echo times, which are preferred for the detection of metabolites exhibiting multiplet resonances. Therefore, quantitation procedures and results should always be inspected by an experienced spectroscopist to exclude misinterpretation.

While at present every MR scanner provides at least basic sequences for MRS acquisition, to our knowledge none of commercial scanners provides robust, reliable and validated quantitation software. Therefore, for metabolite quantitation, third party software [11–13] is most commonly used. Obviously, no software can quantify an arbitrary spectrum automatically without first obtaining appropriate prior knowledge and constraints [14]. This need is satisfied differently by the various software products available. Whereas for LCModel [12] this information is custom made [13], TARQUIN offers a set of predefined constraints and for the most typical acquisition methods the prior knowledge is simulated with an idealized NMR physics model.

jMRUI [11] another widely used quantitation software (currently used in more than 3000 laboratories all around the world), is a flexible tool for spectroscopic signal processing and quantitation: it includes several quantitation algorithms such as AMARES (Advanced Method for Accurate, Robust, and Efficient Spectral fitting) [15], AQSES (Automated Quantitation of Short Echo Time MRS spectra) [16] and QUEST (QUantum ESTimation) [17] and various other quantitation methods based on singular value decomposition (SVD) [18]. Moreover, it also contains a choice of data preprocessing algorithms (e.g. water peak removal, phase correction etc.), data visualization and versatile simulation routines based on quantum mechanics for experiment planning [19] and realistic prior knowledge calculation. Thus it allows quantitation of virtually any type of spectrum but for the price: suitable constraints and prior knowledge must be provided by the user, and sometimes found on a trial and error basis.

Due to the high level of complexity of MRS processing in jMRUI, full tracking of operations done with the spectroscopic signals is required for highly reproducible processing [10, 20] and for the automation of quantification using already verified processing steps in both clinical and research environments. Up till now only a very simple history tracking method was used in jMRUI. jMRUI enables the users to retrieve the quantitation-results files corresponding to successive trials via the set-up menu (the result files can be overwritten or not) but the parameters used can be only partly recovered and not in an automatic way (.op files must be saved). The processing history was saved at the user’s request in a batch file, and only for the most recently loaded data. The batch file included no information about the data processed nor about the results obtained. It could be used for batch processing only, and not for the identification of what processing steps had led to a particular result or how a particular data set had been processed in the past. To respond to such needs, a novel method of tracking the data and all processing operations executed has been developed; this article provides its description.

## Implementation

In this paper the following convention will be kept in the database description:

- Names written in capitals (like FILES) are names of database tables.
- Names written in italics (like *ID_file*) are names of columns in the database.
- Names written in capitals in quotes (like “OPEN FILES”) are names of registries.
- Names written in italics in quotes (like “*SAVE*”) are commands stored in the database.

### Database organization

**Fig. 1:**
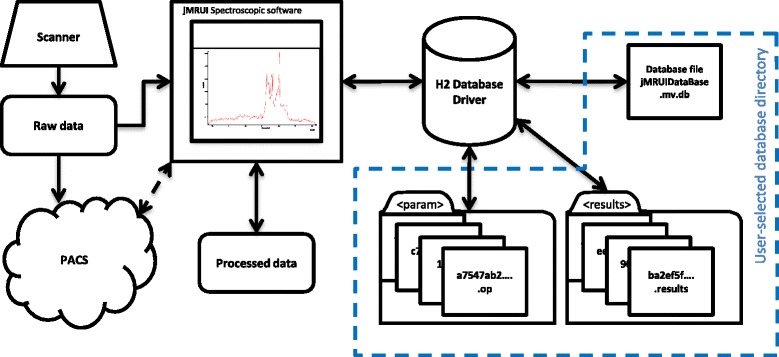
The overall scheme of the data flow history tracking in jMRUI. The scheme shows the flow of the data from the scanner through jMRUI to the database. The database driver connects jMRUI with the file system that stores and retrieves all necessary database-related information. In the user-selected database directory the database file (in this example jMRUIDataBase.mv.db), all parameter files and quantitation results are stored. The parameter files and quantitation results are stored in separate subdirectories. For fast and unambiguous identification, all results and parameter files are renamed with the hash sum of their content

The presented solution uses a database and the file system (Fig. 1) to record all the information necessary to describe the whole processing pipeline from the moment of data loading to saving the quantitation results.

The entire storage system is based on Structured Query Language (SQL) database, file system and a Java class (DBHandling.java) which handles the SQL driver and processes the data before the storage and deals with the file system. The class also connects the jMRUI interface with the stored data and handles the retrieving of the stored information. The class contains registries which are crucial for the functioning of the database and are not stored on the hard disk (e.g. “OPEN FILES” which stores information about all currently open files).

Reliable storage and management of the processing history are provided by a well-established SQL database, and hash sum (also called checksum) was used for the identification of data files. The SQL, a programming language designed for relational databases, is widely applied in the industry because of its high robustness. The H2 database [21] – an SQL database written in Java – was selected for jMRUI as the database engine. One of the advantages of the selected database is that it does not require installation of any additional big software packages; it offers a self-contained single-file installation.

The hash sum is a unique string of numbers and letters of a fixed length that acts as a fingerprint for a particular file. The hash sum is calculated using a hash function, which maps data of an arbitrary size (e.g., the file contents) to a fixed-size code – the hash sum. Because of their ability to provide completely different hash sums even for similar data, hash functions are widely used in cryptography and in digital communication to ensure the integrity of files transmitted.

File identification in the jMRUI database was based on hash function SHA-256. The advantage of such identification over using their locations is that even files which were moved to a different storage location will still be recognized.

### Database SQL tables

**Fig. 2:**
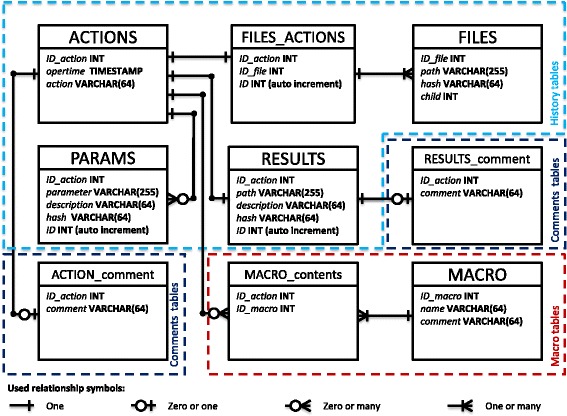
The structure of the jMRUI database. Each table consists of different number of columns (fields). For example in the table ACTIONS there are three columns: *ID_action* (type INT, i.e. integer), *opertime* (type TIMESTAMPS), *action* (type VARCHAR(64), i.e. character string)

Each SQL database is a structure of connected tables. The jMRUI database uses 9 tables. Processing history is stored in 5 tables, comments of actions and results in 2 tables and 2 tables are used for the storage of macros. The structure of the jMRUI database can be found in Fig. 2.

#### ACTIONS

Table ACTIONS records the actions (processing methods) performed on data. It contains 3 columns. In the *ID_action* column an incrementally generated number is stored. The number is used for the action identification in all other tables and enables data linkage between different tables. In the column *opertime* the timestamp of the performed action is saved. Column *action* contains the macro key (short name of each routine) of the corresponding processing method.

#### PARAMS

Table PARAMS contains 5 columns: *ID_action*, *parameter*, *description*, *hash* and the automatically generated and incremented *ID*. Column *ID_action* is used to identify the particular action (and thus processing method) to which the parameters belong. Column *parameter* serves as the storage of the action related parameters; it could be a number, text or a full file name. In the column *description* a comment is always saved, i.e. information about the units used and/or the parameter description. In case the full file name is stored in the *parameter* column as a parameter, the hash sum of the corresponding file for precise file identification is saved in the column *hash.* For organizational purposes there is an additional column (called *ID*), which is automatically incremented.

#### RESULTS

Table RESULTS is used for the storage of information about all quantitation results that were generated in jMRUI. Table RESULTS is very similar to the table PARAMS. The main difference between both tables is that in this table only quantitation results are stored, thus the hash sum of the result file is always computed and stored for reliable result file identification. Column *ID_action* is used to identify the particular action (quantitation method used in this case) that produced the result.

#### FILES

**Fig. 3:**
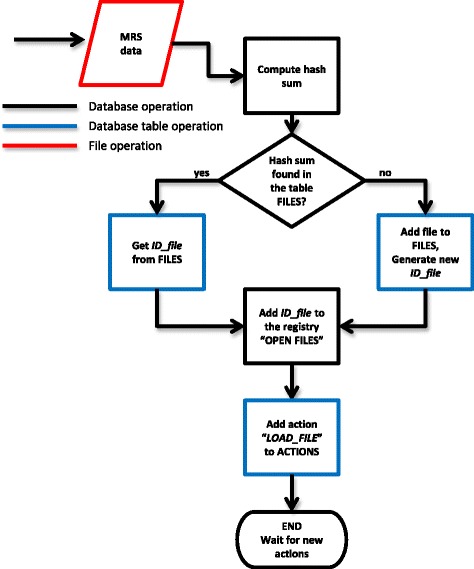
Flow chart of the database operation – registration of a data file loaded. When a file is loaded to jMRUI, the hash sum of the file content is computed. The database searches for the hash sum of the given file in the FILES table; if it is found, the database returns the corresponding file identification number from the *ID_ file* column, otherwise a new entry in the table FILES is created and the new *ID_file* is returned. If more files were loaded, this procedure is repeated for all selected files. All values from *ID_ file* column are stored in the “OPEN FILES” registry and will be kept there until the data file is saved or removed from the jMRUI processing mode. A new entry with “*LOAD_FILE*” value is added to the column action of the table ACTIONS

Table FILES stores information about file handling and it contains 4 columns. During data file loading into jMRUI the hash sum of the file is computed and compared with the table FILES, and if it is not found, a new entry is added to the table FILES. An incrementally generated number is stored in the column *ID_file* and also in the “OPEN FILES” registry of the database (see Fig. 3 for more detailed explanation of the data file workflow)*,* the original location of the data file (full path file name) is stored in column *path*, and the corresponding hash sum of the file is stored in the column *hash*. The column *child* is filled with “-1” by default. If the user decides to save the data processed in jMRUI, a new entry in table FILES will be created with a new value of *ID_file,* the corresponding *path* and the *hash* columns. Column *child* will be filled with the *ID_file* of the file that gave origin to the new file. The original file will be called “parent”.

#### FILES_ACTIONS

Table FILES_ACTIONS provides a link between the tables ACTIONS and FILES. This table contains 3 columns. Whenever a new entry is created in the ACTIONS table, the value of column *ID_action* is inserted also into a new entry in the FILE_ACTIONS table, and *ID_file* column of the FILE_ACTIONS table is filled with the column *ID_file* of the currently processed data (obtained from the “OPEN FILES” registry). There is also an automatically incremented *ID* column, which is used for ordering purposes. This table increases the flexibility of processing tracking by separation of the tables ACTIONS and FILES and it permits to record actions in 1-1 and N-1 relationships.

#### MACRO_contents and MACRO

The information about macros (i.e. sequence of actions done in jMRUI) is stored in two tables. For each created macro a unique number is generated during the creation process and stored in the column *ID_macro* (the same number in both tables). The table MACRO_contents stores sequences of actions of each macro created by the user from a list of actions performed on data in the past. Typically more entries in the table belong to the same macro having the same value in the *ID_macro* column and different values in the column *ID_action.* The values in the column *ID_action* link a macro to the corresponding actions (processing methods and consequently to parameters used in past) in the ACTION table. If the user wants to modify some macro, e.g. to change value of some parameter, this is possible directly in the user interface. The table MACRO stores additional information about saved macros: a unique text name of the macro in the column *name* and user’s comments about the macro in the column *comment*.

#### RESULTS_comment and ACTIONS_comment

Although those two tables are identical and they are used for storage of comments they are separated due to the fact that some actions (mainly quantitation algorithms) also generate results. Both tables contain the *ID_action* and the *comment* columns. ACTIONS_comment contains all comments to ACTIONS, RESULTS_comment is used to store all comments regarding the results.

### Database operation

#### History processing storage

**Fig. 4:**
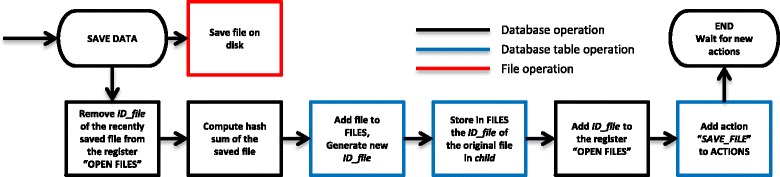
Flow chart of the database operation – registration of a saved data file. If the user decides to save the currently open file, the corresponding *ID_file* number is removed from the “OPEN FILES“ registry. jMRUI saves the file and automatically computes the hash sum of the new file. The computed hash sum and the newly generated *ID_file* are inserted in the table FILES, and the *ID_file* of the original file is saved in the column *child*. The new *ID_file* of the saved file is added to the registry “OPEN FILES”. The file saving operation is recorded in table ACTIONS by adding a new entry with with its *actions* column set to “*SAVE*”

**Fig. 5:**
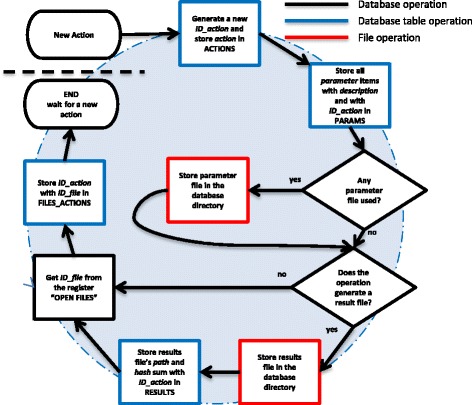
Flow chart of the database operation – registration of actions. If the user processes the loaded data using any preprocessing method in jMRUI, the macro key (short name of each routine), which corresponds to the preprocessing method, is stored in the *action* column of the ACTIONS table. A new action identification number is generated and saved in the column *ID_action* in the ACTIONS table and in all other related tables as shown in the figure. Processing parameters (and if a parameter is a file also the file hash sum) are stored in the PARAMETERS table. The parameter files, if any, are stored in the preprocessing files directory. A new entry is created in the FILES_ACTIONS table linking the processed file (its *ID_file* number obtained from the “OPEN FILES” registry) with the action performed (identified by the action *ID_action* number). If the user decides to quantify the currently loaded data, jMRUI will store also the quantitation result file. The result file is stored in the results file directory and the hash sum of the result file is stored in the table RESULTS

Storing the processing history is performed automatically by the database engine without the user’s interaction. The database engine storage workflow includes several steps as shown in Figs. 3, 4 and 5. The process of data file registration triggered whenever data are loaded/saved in jMRUI is shown in Figs. 3 and 4. The procedure shown in Fig. 5 is triggered each time the user performs any preprocessing or quantitation operation.

In jMRUI database configuration menu the user can choose whether also a text file with the processing history should be generated and saved for each loaded file.

#### History processing extraction

**Fig. 6:**
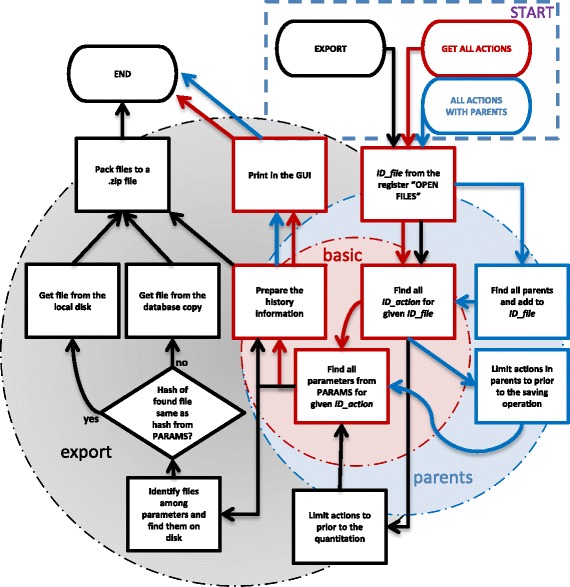
An example of information extraction from the database. The graph is divided into three paths which represent three possibilities of data extraction, “GET ALL ACTIONS” – is a basic database query without sorting and returns all actions done on this particular file, “ALL ACTIONS WITH PARENTS” – is a database query with basic sorting of actions which get information about the origin of the file, “EXPORT” – is a routine which exports the processing history with all parameter files and compresses it into a single.zip file

To extract data from the database a query is needed. Figure 6 demonstrates some examples of extraction process implemented in database.

#### Database interface

Processing history of any data can be obtained by browsing database in the jMRUI database graphical user interface (GUI). Processing history of a loaded data file or a result file can be also obtained by choosing the corresponding menu item command.

#### Menu item commands

The following menu item commands are available for both a data file and a result file loaded:

- Current session – this operation lists all processing steps done in current session. This mode basically provides the same information as we can get from the basic history tracking in jMRUI. In case of opening a result file all steps prior to the quantitation are listed.
- History with parents – this operation lists all processing steps done on the current file including all operations that were done on all files that gave origin to this file.

The following feature is available only in case of loading a data file

- All performed operations – this operation lists all processing steps done on this particular file.

**Fig. 7:**
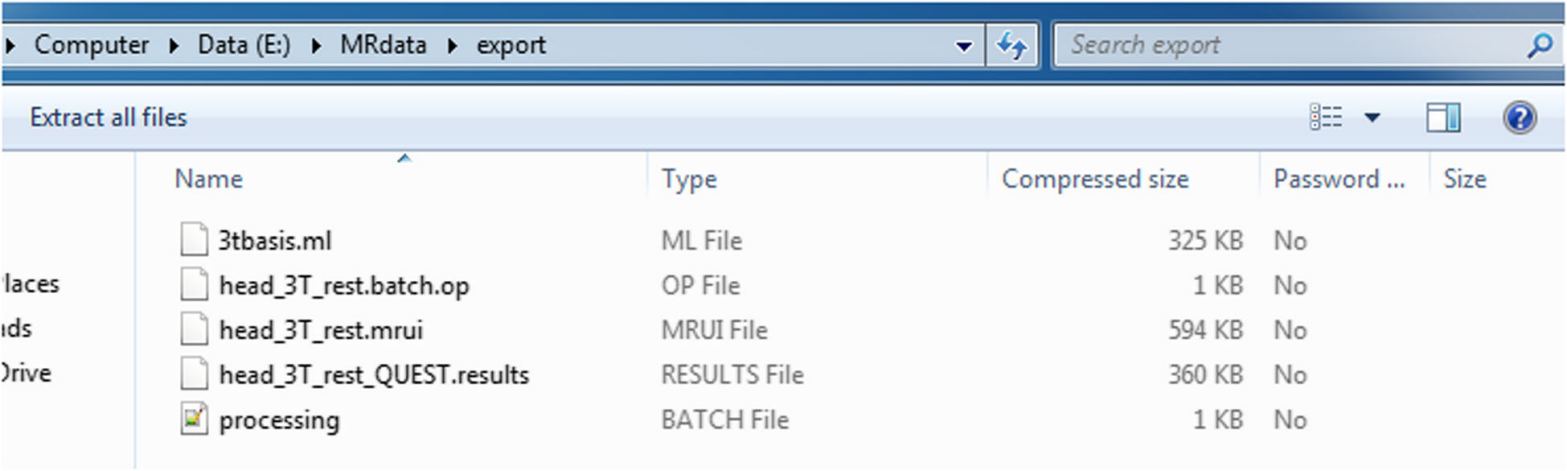
Contents of file generated by “Export all files” used in results mode. In the zip file the user will find all files that were used during quantitation packed to a single zip file. In the generated file the original data file (.mrui file), processing history (file “processing.batch”), QUEST parameter file (.op file) and metabolite basis set list (.ml file) and finally the result file (_QUEST.results file) can be found

The following feature is available in case of loading a result file:

- Export all files – this option does a macro query like in first point and takes all files that were used in the quantitation and pre-processing including the data file and result file. All files are compressed to one zip file and saved in a location selected by the user. An example is shown in Fig. 7.

**Fig. 8:**
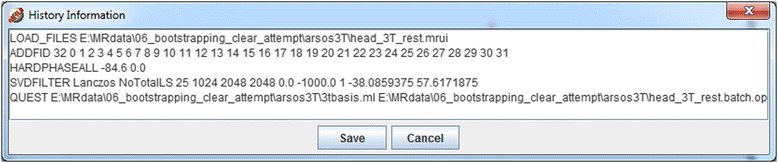
History information obtained from Result mode File. 1D window contains a simplified browser of processing history. The macro obtained in this way can be saved as text file by clicking on “Save”

An example of a history list is shown in Fig. 8.

#### Database graphical user interface (GUI)

**Fig. 9:**
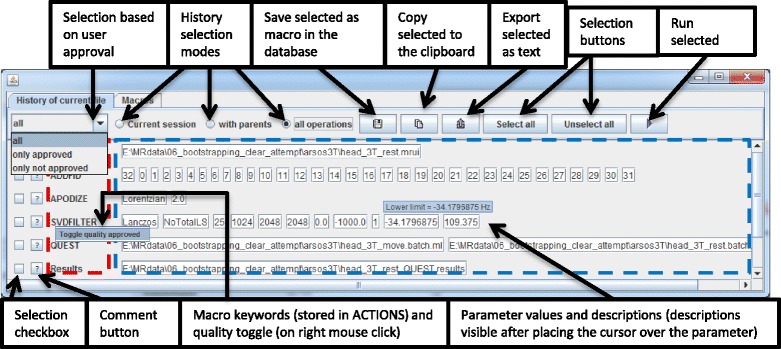
GUI with description of its elements. Main features are described. This GUI can be useful in case of browsing complex processing history

Apart from using the menu item commands, user has possibility to browse the history using graphical user interface. GUI was designed using the Model-View-Controller design pattern. The GUI window and description of functionalities is shown in Fig. 9. The GUI is available as jMRUI Custom Plugin – in the main bar Custom/HistoryGUI.

GUI enables user to:

- Browse history
- Comment an action (a processing method), and classify it as successful or failed attempts.
- Approve an action and later filter it by all actions/only approved/only not approved
- Select actions, modify parameters and run them on loaded data.
- Save selected actions into a macro in the database. The macro can be also exported on demand as a text file any time later. In this way the user can execute a macro (saved actions) with or without modifying parameters on any data in future.
- Copy selected actions as a plain text to clipboard.

### Implementation notes

**Fig. 10:**
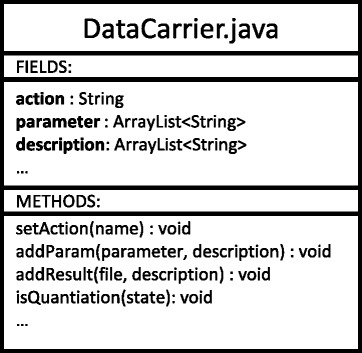
Diagram of the class DataCarrier. In the method histo() the programmer has to create a new object of the DataCarrier class, set its variables such as action keyword, all parameters, short description of parameters, unit if applicable etc. and pass the object created to the database

jMRUI is plugin based software [11]. Each plugin implements one processing method (action). After the data are processed by the plugin the jMRUI Kernel calls the obligatory plugin method histo() that is supposed to pass information about the action performed to the database via newly created object of the class DataCarrier. The class DataCarrier is described in Fig. 10. An example of source code of the method histo() for a quantitation plugin is presented below (Table 1).

**Table 1:** Example of history tracking for a custom made quantitation plugin. In the line 1 an object of DataCarrier class is created. In the line 2 the action keyword is obtained from the plugin property text file and set into the variable action. In the line 3 and 4 parameters (in this case metabolite basis set and parameter file) are set with a brief description. Line 5 enables the quantitation flag (in case of other types of plugins this line should be omitted). Finally the line 6 sends the recently created object to the database. The rest of the process is done in the background and the plug-in programmer does need to care about it

DataCarrier d1 = new DataCarrier();	
d1.setAction(getShortName());	
d1.addParam(new File(metaboliteBasisSetFile), "Metabolite list file = ?");	
d1.addParam(new File(paramFile), "Overall phases file = ?");	
d1.isQuantitation(true);	
mrui.addHistoryStateDB(d1);	

## Results

The main reason of MRS data processing is to quantify the data, which may be a complex process. Due to the fact that jMRUI provides flexible quantitation methods this process may require several attempts based on trial and error method. Moreover the plug-in architecture in jMRUI enables the user to develop new quantitation or processing methods. Those two features show that there is a need for a tool which would track automatically all operations done by the user for later analysis or testing. The delivered tool should provide a user-friendly interface that would enable user to sort and extract easily desired information.

Therefore the database storing all processing steps, parameters and files was developed for jMRUI. The implemented jMRUI database is a tool that can significantly help the user to track the processing history performed on data in jMRUI. The created tool is oriented to be user-friendly, robust and easy to use. For the common user all tracking operations are invisible as they are performed in the background. The database GUI allows the user to browse the whole processing history of a selected file and learn e.g. what processing lead to the results, where the original data are stored, to obtain the list of all processing performed on spectra. The database user interface provides the possibility to create a database instance per study, thus the database size should not pose a problem. The documented history can be used for automation of MRS data processing by generation of macros and for ensuring reproducibility by storing processing protocols together with processed data. It can serve as a database of all processing steps ever performed in jMRUI. The proposed database system could become the scientist’s diary, or a bug tracking tool for software developers. One of the added values of the proposed solution comparing with the earlier version of jMRUI when only the current session history could be stored in a text file is the possibility of getting history information about quantified data. There is a possibility of recovering all processing steps that were used in order to obtain a given results file (with pre-processing macro, and all parameter files) and packing it to a single zip file. This option is especially useful for users that need to recover processing history of their results after some time or share it with other researchers.

The proposed database tracking system includes a new approach to the identification of files, which are identified by the hash sum of the content, and therefore the files will be correctly identified regardless of their location on the disk. In the database GUI user can obtain precise time and date of each operation, get detailed information about stored parameters with short description. The proposed tool provides a possibility of commenting all actions and let the user to select if this particular action was successful or not. Later the user interface can sort the actions based on this quality information. From the development and maintenance point of view all described database functionalities can be used in further jMRUI debugging and improvements. With the history tracking database if the user finds any bug and would like to share this information with the development team the database can generate a pack with all necessary files, parameters and processing history.

We believe that this jMRUI enhancement could bring an important increase of reproducibility in data processing and popularize NMR Spectroscopy in research and clinical environment.

## Discussion

Generally, there are at least three possible approaches to storing the processing history. The first one is to create a text file (macro) that accompanies the original data and the results of the processing. This approach offers basic processing tracking and is not suitable for complex data processing and extensive studies. The authors decided to add this approach to the implemented history tracking system as an auxiliary system.

Another possibility would be to store the processing history directly in the data file as suggested by Mocioiu et al. [22]. They developed a XML interface for jMRUI that stores the processing history and exports it together with data in XML format. This solution was however mainly designed to automate data preprocessing for classification, and offers the storage only of a limited number of listed preprocessing routines and in the fixed order. We found this approach not suitable for complex studies.

The third possibility was to use a database linked with the jMRUI. The authors decided to use this approach which is far less invasive and more robust than the two mentioned approaches and also allows recovering all processing steps ever done in jMRUI. SQL database was designed with certain level of redundancy that makes the overall database more flexible. Other important factor which was taken into account was to provide full compatibility of the database with future releases. Structure of the database enables to add new methods and functionalities in the database engine without changing the structure of tables and thus without changing the database file format.

Thus in future the following improvements can be easily added

- The database file editing could be partially restricted – the user interaction could be limited to just adding new entries without removing and editing the already existing contents in any external application.
- The database could be placed on a centralized server after a few small changes in the database engine.
- The database file could be encrypted - if there is such a necessity, the security of the database can be increased.

Since jMRUI will receive soon a new MR Spectroscopic Imaging (MRSI) interface, the current database version may need some additional adaptation once the new version of MRSI interface is released.

## Conclusions

The jMRUI database system presented helps significantly to tracking of all operations done by the user, it helps to better organize the processing history. The novel approach for file identification guarantees efficient file handling. The implemented system provides a user-friendly GUI several history retrieval operations built-in directly in jMRUI, it increases the reproducibility and documentability of all spectroscopic processing. This functionality provides the user a robust system which could be used as a scientist’s diary registering all data operations performed.

## Availability and requirements

Project name: *jMRUI database*

Project home page: http://www.jmrui.eu

Operating system(s): Windows, Linux

Programming language: Java

License: Free of charge for academic institutions and hospitals (subject to approved registration)

## Abbreviations

MRI: Magnetic resonance imaging
MRS: Magnetic resonance spectroscopy
NMR: Nuclear magnetic resonance
SQL: Structured query language

## References

[1] Oz G, Alger JR, Barker PB, Bartha R, Bizzi A, Boesch C (2014). Clinical proton MR spectroscopy in central nervous system disorders. Radiology.

[2] Govindaraju V, Young K, Maudsley AA (2000). Proton NMR chemical shifts and coupling constants for brain metabolites. NMR Biomed.

[3] Bendahan D, Mattéi JP, Kozak-Ribbens G, Cozzone PJ (2002). Non-invasive investigation of muscle diseases using 31P magnetic resonance spectroscopy: potential in clinical applications. Revue Neurologique.

[4] Mescher M, Merkle H, Kirsch J, Garwood M, Gruetter R (1998). Simultaneous in vivo spectral editing and water suppression. NMR Biomed.

[5] Bottomley PA (1987). Spatial Localization in NMR Spectroscopy in Vivo. Annals of the New York Academy of Sciences.

[6] Frahm J, Klaus-Dietmar Merboldt, Hänicke W (1987). Localized proton spectroscopy using stimulated echoes. J Magn Reson.

[7] Garwood M, DelaBarre L (2001). The return of the frequency sweep: designing adiabatic pulses for contemporary NMR. J Magn Reson.

[8] Scheenen TW, Klomp DW, Wijnen JP, Heerschap A (2008). Short echo time 1H-MRSI of the human brain at 3 T with minimal chemical shift displacement errors using adiabatic refocusing pulses. Magn Reson Med.

[9] Mlynárik V, Gambarota G, Frenkel H, Gruetter R (2006). Localized short-echo-time proton MR spectroscopy with full signal-intensity acquisition. Magn Reson Med.

[10] Kreis R (2004). Issues of spectral quality in clinical 1H-magnetic resonance spectroscopy and a gallery of artifacts. NMR Biomed.

[11] Stefan D, Di Cesare F, Andrasescu A, Popa E, Lazariev A, Vescovo E, Strbak O, Williams S, Starcuk Z, Cabanas M, van Ormondt D, Graveron-Demilly D. Quantitation of magnetic resonance spectroscopy signals: the jMRUI software package. Meas. Sci. Technol. 2009;20:104035.

[12] Provencher SW (1993). Estimation of metabolite concentrations from localized in vivo proton NMR spectra. Magn Reson Med.

[13] Wilson M, Reynolds G, Kauppinen RA, Arvanitis TN, Peet AC (2011). A constrained least-squares approach to the automated quantitation of in vivo (1)H magnetic resonance spectroscopy data. Magn Reson Med.

[14] Graveron-Demilly D (2014). Quantification in magnetic resonance spectroscopy based on semi-parametric approaches. MAGMA.

[15] Vanhamme L, van den Boogaart A, Van Huffel S (1997). Improved method for accurate and efficient quantification of MRS data with use of prior knowledge. J Magn Reson.

[16] Poullet JB, Sima DM, Simonetti AW, de Neuter B, Vanhamme L, Lemmerling P, Van Huffel S (2007). An automated quantitation of short echo time MRS spectra in an open source software environment: AQSES. NMR Biomed.

[17] Ratiney H, Sdika M, Coenradie Y, Cavassila S, van Ormondt D, Graveron-Demilly D (2005). Time-domain semi-parametric estimation based on a metabolite basis set. NMR Biomed.

[18] Pijnappel WWF, van den Boogaart A, de Beer R, van Ormondt D (1992). SVD-based quantification of magnetic resonance signals. J Magn Reson.

[19] Starcuk Z, Starcukova J, Strbak O, Graveron-Demilly D (2009). Simulation of coupled-spin systems in the steady-state free precession acquisition mode for fast magnetic resonance (MR) spectroscopic imaging. Meas Sci Technol.

[20] By: in ’t Zandt, H; van Der Graaf, M; Heerschap, A, Common processing of in vivo MR spectra. NMR in biomedicine doi: 10.1002/nbm.707 10.1002/nbm.707 11410940

[21] H2 Database Engine http://www.h2database.com/html/main.html 15 July 2016

[22] Mocioiu V, Ortega-Martorell S, Olier I, Jablonski M, Starcukova J, Lisboa P, Arús C, Julià-Sapé M (2015). From raw data to data-analysis for magnetic resonance spectroscopy – the missing link: jMRUI2XML. BMC Bioinformatics.

